# Enhancement of porous asphalt mixtures modified with various fibers and ethylene–vinyl acetate polymer

**DOI:** 10.1038/s41598-024-65615-y

**Published:** 2024-07-06

**Authors:** Kareem G. Nazmey, Mohammed S. Eisa, Ahmed Gamal M. Morsi, Ahmed S. Debaiky

**Affiliations:** Civil Engineering Department, Benha Faculty of Engineering, Benha University, Benha, Egypt

**Keywords:** Porous asphalt mixtures, Open-graded friction course (OGFC), Cellulose fibers, Glass wool fibers, Ethylene–Vinyl acetate (EVA) polymer, Performance tests, Civil engineering, Structural materials

## Abstract

Porous asphalt mixture is conventional hot mix asphalt (HMA) with substantially decreased fines, which produces an open-graded mixture that enables the water to flow through an interconnected void space. Porous asphalt is a permeable system that has a lot of benefits. However, because of its open structure, the durability of this mixture decreases, and both its stability and resilient modulus are much lower compared to the dense conventional asphalt mixtures. Also, the high void percentage may lead to an increase in the draindown proportion. Fibers (cellulose or mineral) and polymer-modified binders are recommended for porous asphalt mixtures, especially in hot and moderate climates. The objective of this study is to improve the porous asphalt mixture's performance by using ethylene–vinyl acetate (EVA) polymer-modified bitumen. Two types of fibers (cellulose fibers and glass wool fibers) were used, separately to determine the control mixture. Four different proportions of EVA polymer were added to the bitumen (1%, 2%, 3%, and 4%) and Scanning Electron Microscopy (SEM) was used for better investigating of the bitumen microstructure, then The Marshall mix design was used to determine the optimum EVA content (OEC) for the porous asphalt mixture. Several performance tests were conducted to investigate the characteristics of the porous asphalt mixture, such as the infiltration rate, binder draindown, the wheel track and the cantabro abrasion tests. The findings of the study conclude that the addition of EVA polymer to the porous asphalt mixtures enhances the performance as it increases stability by 20.8% and the infiltration rate by 20.6%. It decreases binder draindown proportion by 33.3%, cantabro abrasion loss by 25.1% and the rut depth at 5,000 cycles and 10,000 cycles by 29.8% and 19.7%, respectively.

## Introduction

Porous asphalt pavement is substitute paving surface that enables rainwater runoff to permeate through the pavement surface voids into a crushed stone reservoir beneath, where it can be temporarily held and/or infiltrated. Porous asphalt pavement is a permeable system which has a lot of benefits, it is used to reduce drainage system infrastructure and its costs, mitigate the occurrence of hydroplaning on roadways and the accumulation of standing water on pedestrian walkways and increase vehicle safety. It’s also used to lessen wet-weather glare, minimize the risk of damage caused by freeze/thaw conditions such as the formation of cracks compared to traditional pavement and reduce tire-pavement noise^1,2^.

Porous asphalt mixture typically consists of conventional hot mix asphalt (HMA) or warm mix asphalt (WMA) with substantially decreased fines, producing an open-graded mixture that enables water to flow through an interconnected void space. The porosity of the asphalt surface normally falls in the range of 18% to 25%. Porous asphalt mixture is suitable for usage in most climate conditions where the conventional mixture is appropriate^1^. However, the durability of the open-graded mixture is significantly decreased because of its open structure, and it has significantly lower stability and resilience modulus than dense conventional asphalt mixtures^3^. Also, porous asphalt mixtures with low fillers, a high void percentage, and high binder content may lead to an increase in draindown proportion, i.e., draining the bitumen from the mixture during the transportation and laying process.

Significant research efforts have been done to enhance the durability and strength of porous asphalt mixtures and decrease the draindown percentage. Adding fibers to the porous asphalt mixtures is desirable to significantly reduce draindown and make a durable open-graded friction course; either cellulose fibers or mineral fibers can be used^2^. Fibers are used to increase the bitumen binder content of the porous asphalt mixtures and thus lead to increase film thickness, which leads to better durability^4^. Furthermore, for enhanced durability of porous asphalt mixtures, it is recommended to use bitumen with 60/70 penetration grade and polymer-modified bitumen, particularly in hot and moderate climates with medium to heavy traffic^5^. The characteristic of asphalt binder is a crucial factor affecting the porous asphalt mixtures performance. Currently, the utilization of polymer modified bitumen has become a common procedure to enhance the performance of porous asphalt^6^.

Mineral fibers are very common addition to the porous asphalt as it affects its workability and its overall mechanical performance, but the primary function of mineral fibers is to minimize the possibility of asphalt binder draindown^6^. An investigation was conducted to examine the impact of basalt fiber on enhancing the performance of porous asphalt mixture. It was determined that at small addition of basalt fiber (0.2% and 0.4%), the performance was improved, especially the strength properties which lead to an improvement in the service life of the mixtures, but at fiber addition more than 0.4%, the strength was reduced because the fiber volume reduced the adhesion between aggregate and bitumen^7^. Another investigation was conducted to evaluate the performance porous asphalt mixtures using clay brick dust as mineral filler. The results showed that the modified mixtures enhanced the binder draindown as it decreased from 0.3% for the conventional porous mixtures to 0.08%, but the presence of clay brick dust resulted in a reduction in the ability of resisting the rutting^8^. A Bottom Ash boiler was investigated as filler to the porous asphalt mixture with cement and it was concluded that a 6% asphalt content with a variation of filler 50% cement and 50% bottom ash boiler met the Australian Asphalt Pavement Association AAPA 2004 Specifications^9^. Porous asphalt mixture stabilized by cellulose fibers was investigated and it was concluded that it had the same performance as porous asphalt mixture stabilized by mineral fibers and with addition of 0.3% cement dust, it could present a higher performance^10^.

A study on thermoplastic resin modified porous asphalt mixture was done to evaluate its performance and the effect of different preparation process was also studied. Three preparation methods, including the dry process (DP), wet process (WP), and dry/wet mixing process (MP), were employed. The findings indicated that resin-modified asphalt prepared using DP and MP, exhibits lower resistance to high-temperature deformation and low-temperature cracking compared to the WP. The dynamic stability of the resin high viscosity modifier RHVM asphalt mixture, produced by using the DP and MP methods, exhibited a drop of 17.3–18.2% compared to the RHVM (WP) asphalt mixture. Indirect tensile strengths for all mixtures were within the acceptable specification range^11^. Characteristics of porous asphalt mixtures modified with Styrene–Butadiene–Styrene (SBS) polymer were investigated. Two types of SBS polymeric molecular chains (1101 AT and D0243) were used to modify a 50/70 neat asphalt binder which led to generate 60/85 E and highly modified asphalt matrixes (HiMA), respectively. The results showed that HiMA matrix exhibited a better long-term rheological and mechanical performance and greater rutting resistance, but because the matrix had a high dynamic viscosity, it implied a small reduction to the porosity of the mixture^12^. Several recent studies have primarily studied the utilization of various fibers to improve the effectiveness of porous mixtures. Additionally, some studies have explored the usage of elastomers such as SBS. However, there has been insufficient research conducted on the application of plastomer-modified binder in porous mixtures.

Many polymer-modified binders were used with conventional dense mixtures, and a considerable research has been done to study their performance. However, the utilization of polymer-modified binder with porous asphalt mixtures, especially the plastomers, is not as common as its use with conventional dense mixtures. The objective of this study is to enhance the performance of porous asphalt mixtures by utilizing a polymer-modified binder, which leads to an improvement in service life. In this study, ethylene–vinyl acetate polymer (EVA polymer) was used with 60/70 bitumen. EVA polymer is the most commonly used plastomer and was one of the first polymers to be effectively utilized in asphalt applications^13^. It mainly stiffens the binder, enhancing the asphalt's ability to resist rutting and traffic loading, especially in hot weather. One of the appealing characteristics of utilizing EVA is that it efficiently functions as a diluent at high mix temperatures, i.e., primarily above 100 °C^13^. Another important characteristic of EVA is that it completely melts and disperses into the bitumen^14^. Two types of fibers (cellulose fibers and glass wool fibers) were used, separately to determine the control mixture. Then, four different proportions of EVA polymer were added to the bitumen (1%, 2%, 3%, and 4%) to determine OEC. The microstructure of EVA-modified bitumen was studied using SEM analysis. Then, many tests were performed to investigate the performance of the porous asphalt mixtures, such as Marshall stability, binder draindown, permeability, cantabro abrasion loss, and rutting resistance.

## Materials

The materials used in this study were open graded aggregates, Bitumen (60/70) as binder, glass wool fibers as mineral fibers, Cellulose fibers as natural fibers and Ethylene–vinyl acetate polymer (EVA Polymer) as an additive to the bitumen. Many tests were conducted to investigate the validity of these materials.

### Aggregates

**Figure 1:**
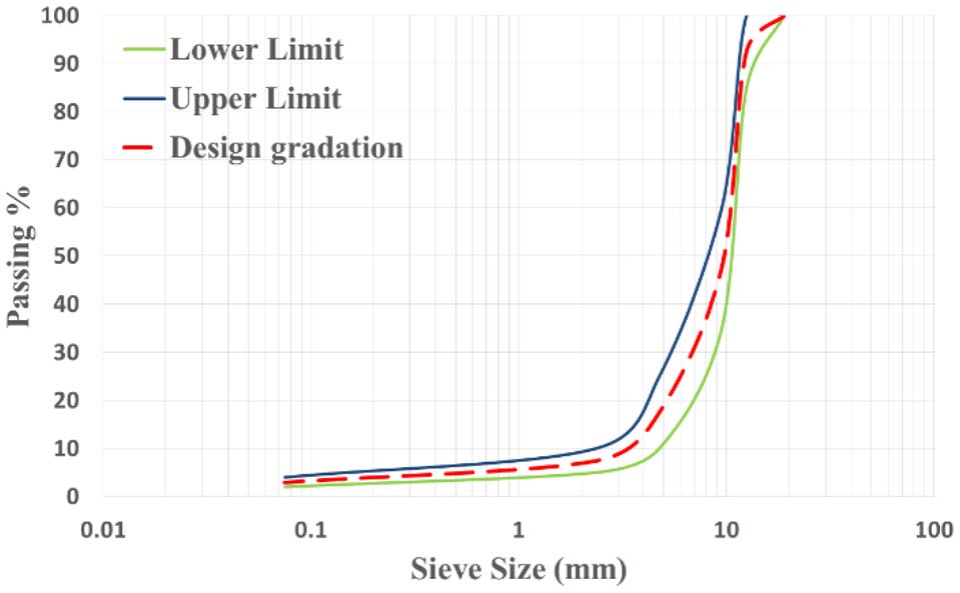
Aggregate design gradation compared to the specification limits.

The aggregates used to prepare the porous asphalt mixtures were crushed dolomite stone as coarse aggregate, clean crushed and natural sand as fine aggregate, and fillers. The aggregates used were open graded. Figure 1 show the aggregate design gradation used in the experimental program with the gradation limits of the open graded-friction course according to National Asphalt Pavement Association NAPA IS 115 (2002)^2,15^. Table 1 shows the properties of the aggregate used.

**Table 1:** Properties of the aggregate used.

Test	Results		Egyptian standards	Standard test method
	Coarse	Fine		
L.A. abrasion (%)	27.04	-	Up to 40%	ASTM C131
Water absorption (%)	1.37	1.12	Up to 5%	ASTM C127 ASTM C128
Bulk specific gravity	2.62	2.53	–	
SSD specific gravity	2.65	2.56	–	
Apparent specific gravity	2.71	2.61	–	
Flakiness index (%)	8.91	–	Up to 10%	ASTM D4791
Elongation index (%)	3.80	–	Up to 10%	ASTM D4791

### Bitumen

The asphalt binder was bitumen (60/70), as it is the most widely used type in Egypt. It came from a refinery located in Suez City, Egypt. Table 2 shows the properties of the bitumen used.

**Table 2:** Properties of the Bitumen used.

Test	Result	Egyptian standards	Standard test method
Penetration (0.1 mm)	64	60–70	ASTM D5
Softening point (°C)	51	45–55	ASTM D36
Flash point (°C)	+ 250	≥ 250	ASTM D92
Kinematic viscosity (C.St)	428	≥ 320	ASTM D2170
Specific gravity	0.99	–	ASTM D70
Dynamic viscosity (poise)	4.24	–	ASTM D4402

### Fibers

**Figure 2:**
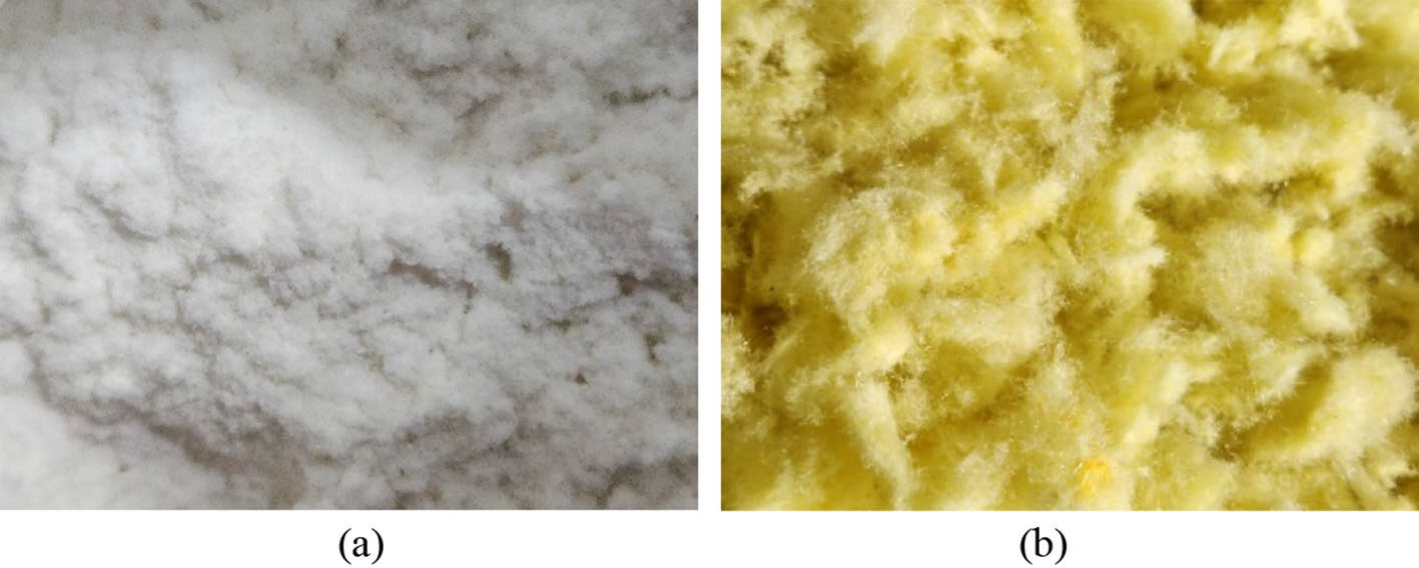
Fibers used: (**a**) cellulose fibers. (**b**) Glass wool fiber.

Fibers are added to the porous asphalt mixtures in accordance with the recommendations of NAPA IS 115 (2002)^2^. Two types of fibers were used separately. The first one is cellulose fiber as a natural fiber and it presents itself as a more environmentally sustainable material. The second one is glass wool fiber as mineral fiber. Tables 3 and 4 show the properties of cellulose fiber and glass wool fiber, respectively. Figure 2 shows the cellulose fiber and the glass wool fiber used.

**Table 3:** Properties of the Cellulose fibers used.

Property	Result
Fiber type	Wood pulp
Fiber length (mm)	0.063–0.5
Cellulose content (%)	98.2
Specific gravity	0.15–0.2

**Table 4:** Properties of the glass wool fibers used.

Property	Result
Melting point (°C)	> 450
Average fiber diameter (µm)	3–5
Specific gravity	0.1
Flammability	Not inflammable
Color	Yellow

### Additive

**Figure 3:**
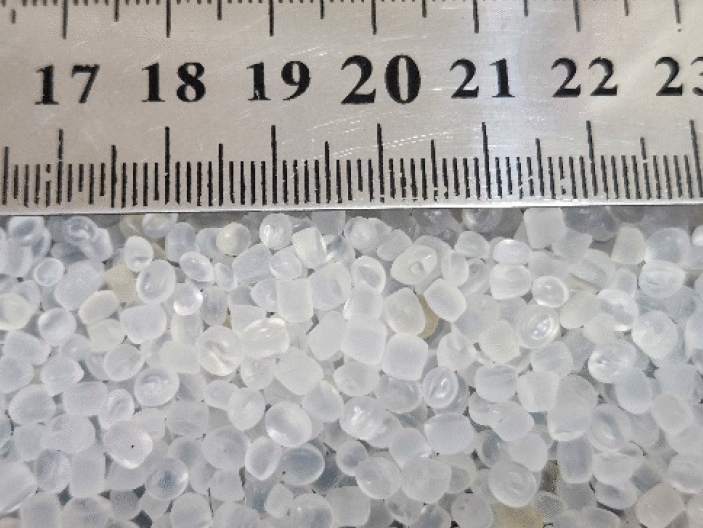
EVA polymer.

To get a polymer-modified binder, EVA polymer was added to the bitumen. Figure 3 shows the EVA polymer used. Table 5 shows the properties of the EVA polymer as it came from the data sheet of the producer^16^.

**Table 5:** Properties of EVA polymer.

Property	Result	Test method
Melt flow rate (g/10 min)	2.5	ISO 1133
Vinyl acetate content (%)	19	Internal method
Density (g/cm^3^)	0.941	ISO 1183
Melting point (°C)	83	Internal method
Brittleness temperature (°C)	< − 80	ASTM D 746
Vicat softening point (°C)	58	ISO 306/A
Tensile stress at yield (Mpa)	4.5	ISO 527
Flexural modulus (Mpa)	40	ISO 178

## Experimental program

**Figure 4:**
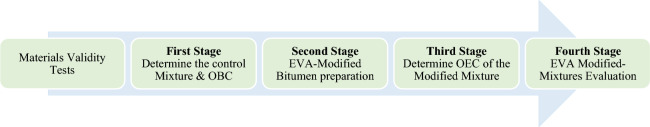
Experimental program stages chart.

The experimental program of this study went through four stages. Figure 4 illustrates all stages of the experimental program in this study.

### First stage

The first stage was dedicated to determining the optimum bitumen content (O.B.C.) for the control mixture. Fibers are used in porous asphalt mixtures to reduce draindown for a durable open graded-friction course^2^. Two types of fibers (natural and mineral fibers) were used in the control mix separately, as both of them are effective in porous asphalt mixtures^2,10,17^. Cellulose fibers with a percentage of 0.3% of the total mix and glass wool fibers with a percentage of 0.4% of the total mix were used as recommended by NAPA IS 115 (2002)^2^. Five different cellulose porous asphalt mixtures were made with bitumen contents (4%, 4.5%, 5%, 5.5%, and 6%), and another five different glass wool porous asphalt mixtures were made with bitumen contents (4%, 4.5%, 5%, 5.5%, and 6%). All mixtures were prepared according to the Marshall mix design method (ASTM D 1559)^18^. 50 blows with a Marshall compaction hammer were applied according to the recommendations of NAPA IS 115 (2002) and AAPA Second Edition April (2004)^2,19^. Then all mixtures were tested by the Marshall apparatus to obtain the stability and flow of each mixture. Then, a comparison was made between the cellulose mixtures and the glass wool mixtures to determine the control mixture for next stages.

### Second stage

The objective of the second stage was to prepare EVA-modified bitumen and do conventional bitumen tests on the prepared samples to determine the properties of EVA-modified bitumen. Five bitumen tests were conducted on the prepared samples: Penetration, Softening Point, Flash Point, Dynamic Viscosity at 135 °C, and Specific Gravity (SG). Then, kinematic viscosity could be calculated from the dynamic viscosity value and SG value by Eq. (1):

1 $$Kinematic viscosity=\frac{\text{Dynamic viscosity}}{\text{Specific Gavity}}$$

Furthermore, by utilizing the findings of the penetration and softening point tests, the EVA-modified bitumen's temperature susceptibility was determined in terms of the penetration index (PI). Temperature susceptibility refers to the variation in the consistency parameter of bitumen in response to changes in temperature. Lower values of the penetration index imply more sensitivity to temperature changes. Asphalt mixtures with a higher penetration index of bitumen exhibit increased resistance to both low-temperature cracking and permanent deformation^20^. The Shell Bitumen Handbook presents a conventional method for computing the value of PI^21^, as demonstrated by Eq. (2):

2 $$PI=\frac{1952-500\text{ Log}\left(\text{Pen}.\right)-20\text{ SP}}{50\text{ Log}\left(\text{Pen}.\right)-\text{SP}-120}$$

where, Pen. is the penetration value at 25 °C (in 0.1 mm increments) and SP is the softening point temperature (in °C) of EVA modified bitumen.

EVA concentrations were chosen as 1%, 2%, 3%, and 4% of the bitumen weight. The base bitumen was heated to a fluid state at a temperature of 170 °C during the preparation process. Subsequently, it was poured into a suitable container, and the EVA polymer was mixed with the bitumen. The EVA-modified bitumen samples were prepared using a shear laboratory-type mixer, which was rotated at a speed of 5000 rpm for a duration of 1.5 h. The mixer was maintained at a constant temperature of 140 °C to ensure a complete and uniform dispersion of EVA in the base bitumen. The microstructure of EVA-modified bitumen was investigated using Scanning Electron Microscopy (SEM) to evaluate the uniformity of dispersion of EVA within the base bitumen. SEM images can offer insights into the distribution of the polymer-rich phase. It also specifies the ideal composition that can maintain the stability of the network between bitumen and polymer^22^.

### Third stage

The third stage was dedicated to determining the optimum EVA polymer content for the modified mixtures (O.E.C.). Four different EVA porous asphalt mixtures were made based on the first stage's findings with different EVA contents (1%, 2%, 3%, and 4%). All mixtures were prepared according to the Marshall Mix design method, as stated previously in stage one.

### Fourth stage

The fourth stage was dedicated to evaluating the modified mixture after determining the O.E.C. and making a comparison between the control mixtures and the modified mixtures. Four different performance tests were conducted on both mixtures.

The first test was the “Determination of Draindown Characteristics Test”. This test method measures the amount of draindown material in an uncompacted asphalt mixture sample and determine if the measured amount of draindown material falls within acceptable ranges. The sample is subjected to high temperatures similar to those experienced throughout the production, storage, transportation, and placement of the mixture. The sample is placed inside a wire basket, which is then positioned upon a plate. The sample, basket, and plate were set in a forced-draft oven and subjected to a preselected temperature for a duration of 1 h. After 1 h, the basket is taken out of the oven, along with the plate, and the mass of the plate is measured. Then, the quantity of draindown material is calculated as shown in Eq. (3). All test procedures were according to (ASTM-D6390)^23^.

3 $$draindown \%=\frac{{M}_{f}- {M}_{i}}{{M}_{t}}$$

where M_f_ = final plate mass; M_i_ = initial plate mass; and M_t_ = initial total sample mass.

The second test was “Surface Infiltration Rate of Permeable Unit Pavement” and its objective was to investigate the capability of porous asphalt mixtures to discharge the falling water to the drainage system. For this experiment, the permeable pavement system has an infiltration ring temporarily sealed to its surface. After the sample is pre-wetted, a given amount of water is added to the ring and the duration it takes for the water to penetrate the pavement is recorded. Then, the infiltration rate is calculated according to Eq. (4). All procedures of the test were according to (ASTM-C1781/1781M)^24^.

4 $$I=\frac{\text{K}*\text{M }}{{D}^{2}*\text{t}}$$

where I = Infiltration rate, mm/h, M = Mass of infiltrated water, kg, D = Inside diameter of infiltration ring, mm, t = time required for measured amount of water to infiltrate the surface, s, and K = 4,583,666,000 mm^3^ s/kg h.

The third test was “The Cantabro Abrasion Test” in order to determine the abrasion loss value of the porous asphalt mixtures using the Los Angeles abrasion machine. This test carried on three Marshall samples by abrading them in the Los Angeles apparatus without the abrasion load (the steel balls) for 300 revolutions and the drum should be turned at 30–33 revolutions per minute. The result of the test is determined using Eq. (5). All procedures of the test were according to (ASTM-D7064/7064M)^25^.

5 $$P=\frac{\text{P}1-\text{P}2}{\text{P}1 } \times 100$$

where P = Cantabro abrasion loss, P1 = initial weight of the sample, and P2 = final weight of the sample.

The fourth test was “Hamburg Wheel-Track Testing of Compacted Hot Mix Asphalt (HMA)” and its objective was to measure the rutting of the porous asphalt specimen. This test involves subjecting a laboratory-compacted specimen of HMA to repeated loading using a reciprocating steel wheel. The sample is immersed in a water bath that is controlled to maintain a specific temperature between 4 and 72 °C^26^. Then, the measurement of the specimen's deformation resulting from the loading of the wheel is conducted. All procedures of the test were according to (AASHTO-T324)^27^.

## Results and discussion

As mentioned earlier, the experimental program went through four stages. Here are the results of each stage:

### First stage

**Figure 5:**
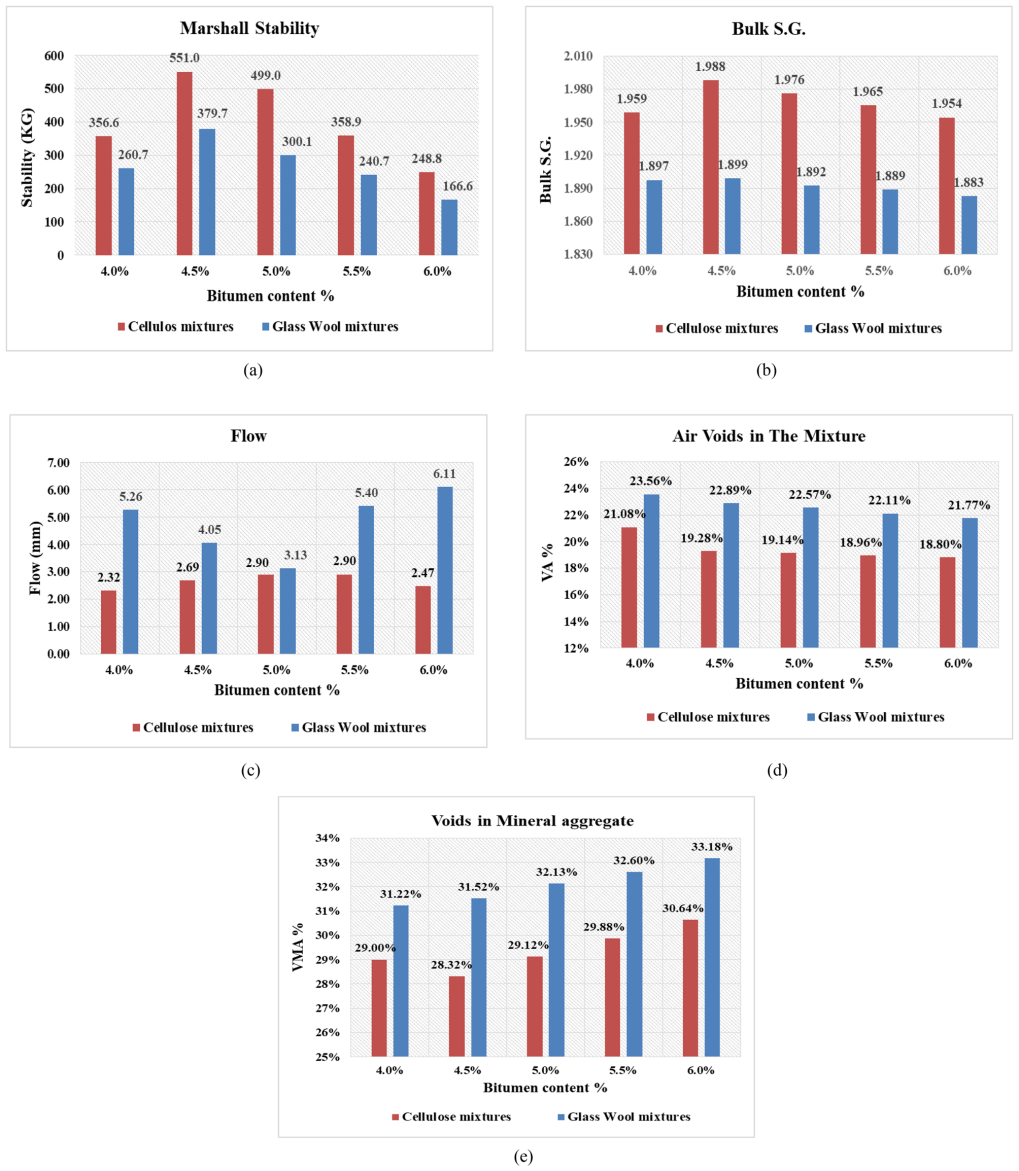
Marshall test results for cellulose mixtures and glass wool mixtures. (**a**) Stability results. (**b**) Bulk specific gravity results. (**c**) Flow results. (**d**) Air voids results. (**e**) Voids in mineral aggregate results.

The objective of the first stage was to compare between the porous asphalt mixtures with 0.3% cellulose fibers and the porous asphalt mixtures with 0.4% glass wool fibers through Marshall Test results and find the O.B.C. for the control mixtures. Figure 5a–e show the Marshall Test results for both mixtures, as they show the relation between the percentages of bitumen content and stability, S.G., flow, VA%, and VMA%, respectively.

**Figure 6:**
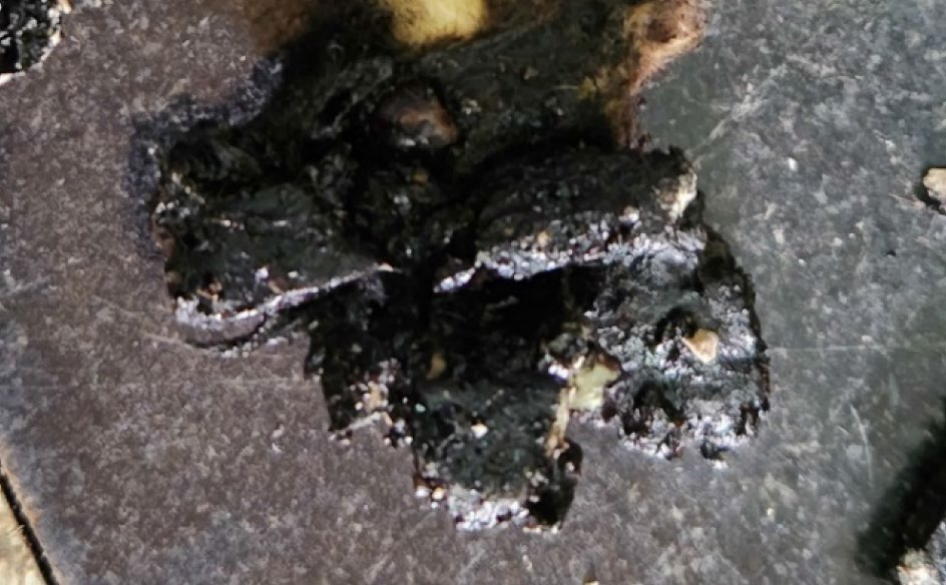
Glass wool fiber clumps found in the mixtures.

From the results, it was found that the stability of the cellulose mixtures reached its peak at 4.5% bitumen, increasing from 356.6 kg at 4% bitumen to 551.0 kg at 4.5% bitumen, then the trend went down. Similarly, the glass wool mixtures stability also reached its peak at 4.5%, increasing from 260.7 kg at 4% bitumen to 379.9 kg at 4.5%, followed by a decline. The cellulose mixtures enhanced the Marshall stability by 45% compared to the glass wool mixtures. However, the glass wool mixtures stability didn’t meet the requirement of NAPA IS115, which is 500 kg minimum^2^, whereas the cellulose mixtures did meet this requirement of NAPA IS115. This happened because of the poor workability of the glass wool in the asphalt mixtures, as it didn’t get fully homogenous in the mixture with the other ingredients. Also, it was found that the glass wool fibers had a tendency to absorb bitumen excessively, so they formed clumps in the mixture, which led to uneven distribution of the fibers and diminished the performance of the mixture. Figure 6 shows the clumps of glass wool fibers found in the mixtures. On the other hand, cellulose fibers exhibited better homogeneity in the mixtures.

The specific gravity of the cellulose mixtures reached its peak at 4.5% bitumen, increasing from 1.959 at 4.0% bitumen to 1.988 at 4.5% bitumen, then decreasing to 1.954 at 6% bitumen. From the flow results, it was found that all cellulose mixtures met the requirement of NAPA IS115, which is 2–6 mm^2^. At 4.5% bitumen, the Marshall flow was 2.69 mm, resulting in a Marshall quotient of 204.8 kg/mm as the stability was 551.0 kg. Air void results indicated that increasing the bitumen in the mixture led to a decrease in the air void percentage, but all cellulose mixtures met the NAPA IS115 requirement of a minimum of 18%^2^. The OBC of the cellulose mixtures was determined based on the previous results and found to be 4.5%, as it gave the highest stability, highest bulk specific gravity value and reasonable flow and air voids percentage.

Table 6 shows the properties of the OBC porous asphalt mixtures with cellulose fibers.

**Table 6:** The properties of OBC porous asphalt mixtures with cellulose fibers.

Property	Result	(NAPA) requirements for OGFC
Stability (kg)	551	500
Flow (mm)	2.69	2–6
Marshall quotient (kg/mm)	204.83	200
Air voids (%)	19.28	> 18
Bulk specific gravity	1.988	–
Voids in mineral aggregate (%)	28.32	–

Eventually, the cellulose mixtures gave better performance than the glass wool mixtures, and it was favorable to use the cellulose mixtures in the next stages as the control mix.

### Second stage

**Figure 7:**
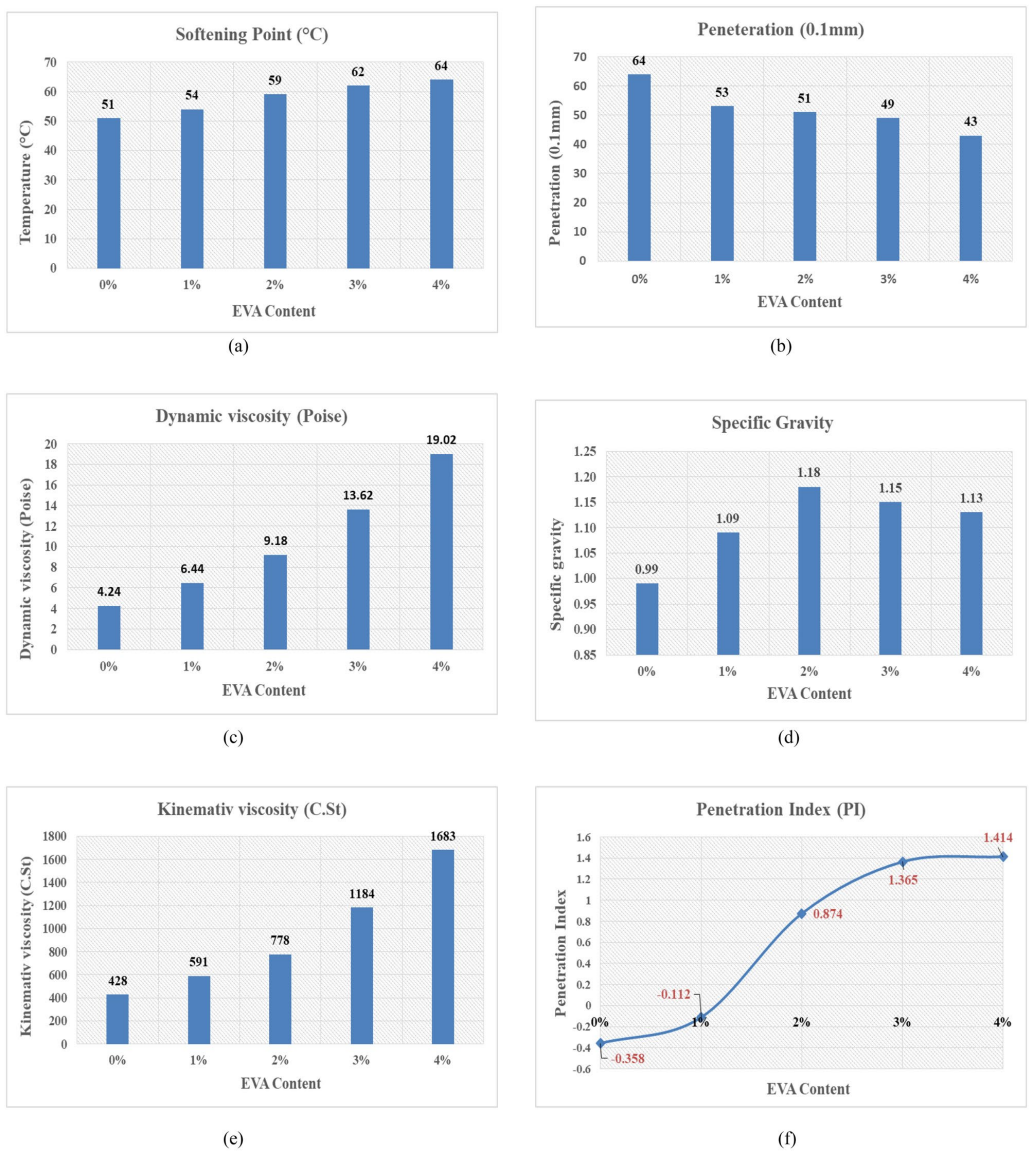
EVA-modified bitumen test results (**a**) softening point values (**b**) penetration values (**c**) dynamic viscosity values (**d**) specific gravity values (**e**) kinematic viscosity values (**f**) penetration index values.

The objective of the second stage was to determine the properties of EVA-modified bitumen. The effect of EVA polymer modification can be seen in Fig. 7a–f. They illustrate the relationship between the percentage of EVA polymer content and softening point, penetration, dynamic viscosity, specific gravity, kinematic viscosity, and penetration index, respectively.

From the results, it was found that the increase in EVA polymer content led to a considerable increase in softening point values and a significant decrease in penetration values, particularly at polymer content of 1%, as the penetration value dropped from 64 for the base bitumen to 53 at polymer content of 1%. The increase in softening point, which is an indicator of bitumen stiffening effect, is favorable, as the bitumen with a higher softening point value may be less susceptible to rutting (permanent deformation)^14^. Additionally, there was a significant increase in the viscosity of the modified bitumen, especially at polymer contents of 3% and 4%, which is favorable as the high viscosity binder can significantly enhance the overall performance of the porous asphalt mixtures^28^. Furthermore, it was found that EVA-modified bitumen reduced temperature susceptibility, as indicated by penetration index PI graph as the value of PI increased from -0.358 for the base bitumen to + 1.414 for 4% EVA polymer content. This might have occurred due to the nature of the EVA polymer, which is a type of plastomer that has a tendency to undergo recrystallization after melting into the bitumen. As a result, it caused the bitumen to become stiffer.

**Figure 8:**
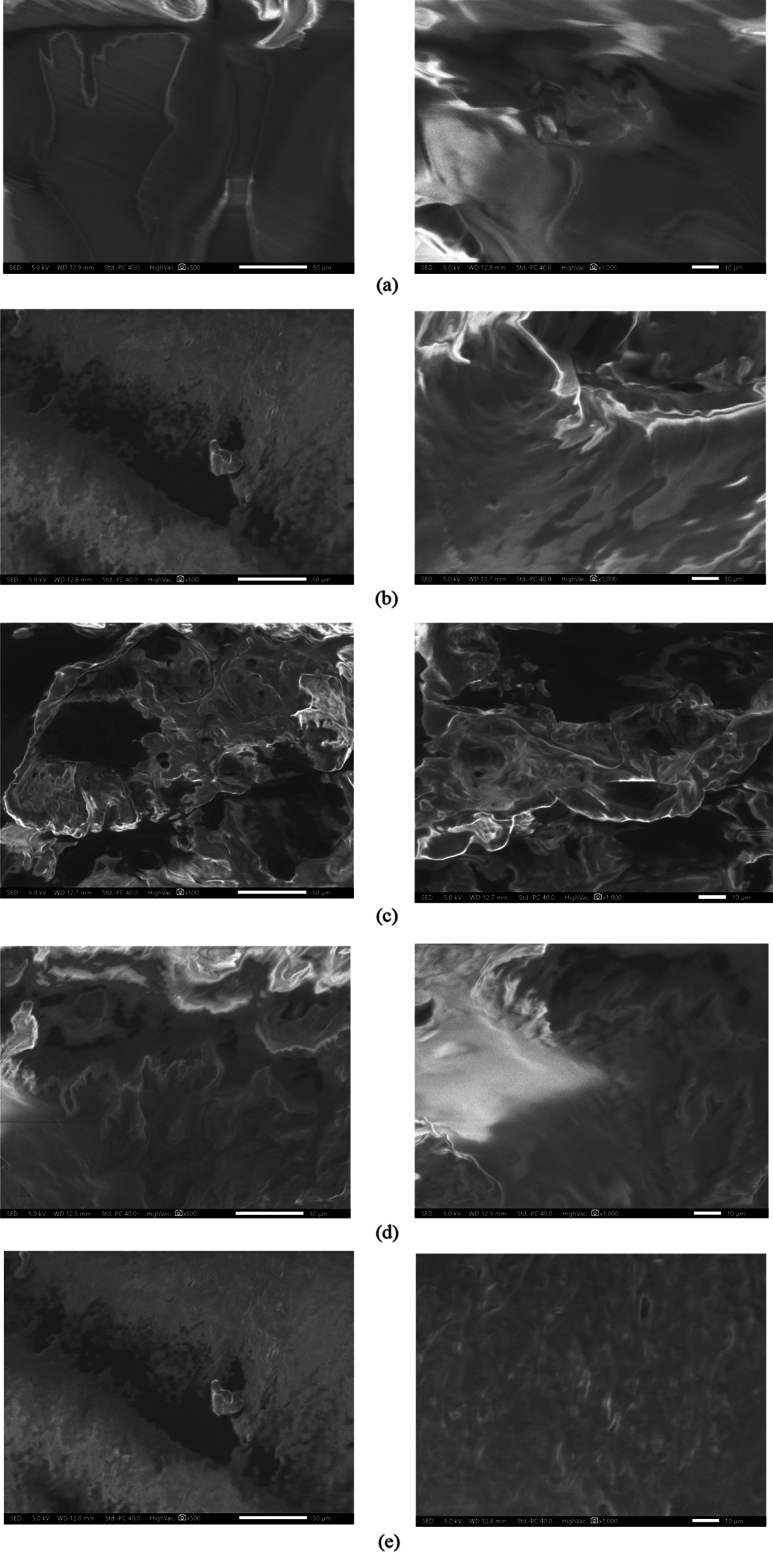
SEM images for (**a**) base bitumen. (**b**) 1%. (**c**) 2%. (**d**) 3%. (**e**) 4% EVA modified bitumen.

Also, in that stage, the microstructure of EVA-modified bitumen was investigated using SEM images to evaluate the uniformity of dispersion of EVA within the base bitumen. Figure 8a–e show the SEM images of base bitumen, 1%, 2%, 3%, and 4% EVA modified-bitumen, respectively. The images sequence shows how bitumen microstructure is affected by EVA polymer and for all concentrations, homogeneity can be clearly seen in terms of surface morphology which coincides with the literature.

### Third stage

**Figure 9:**
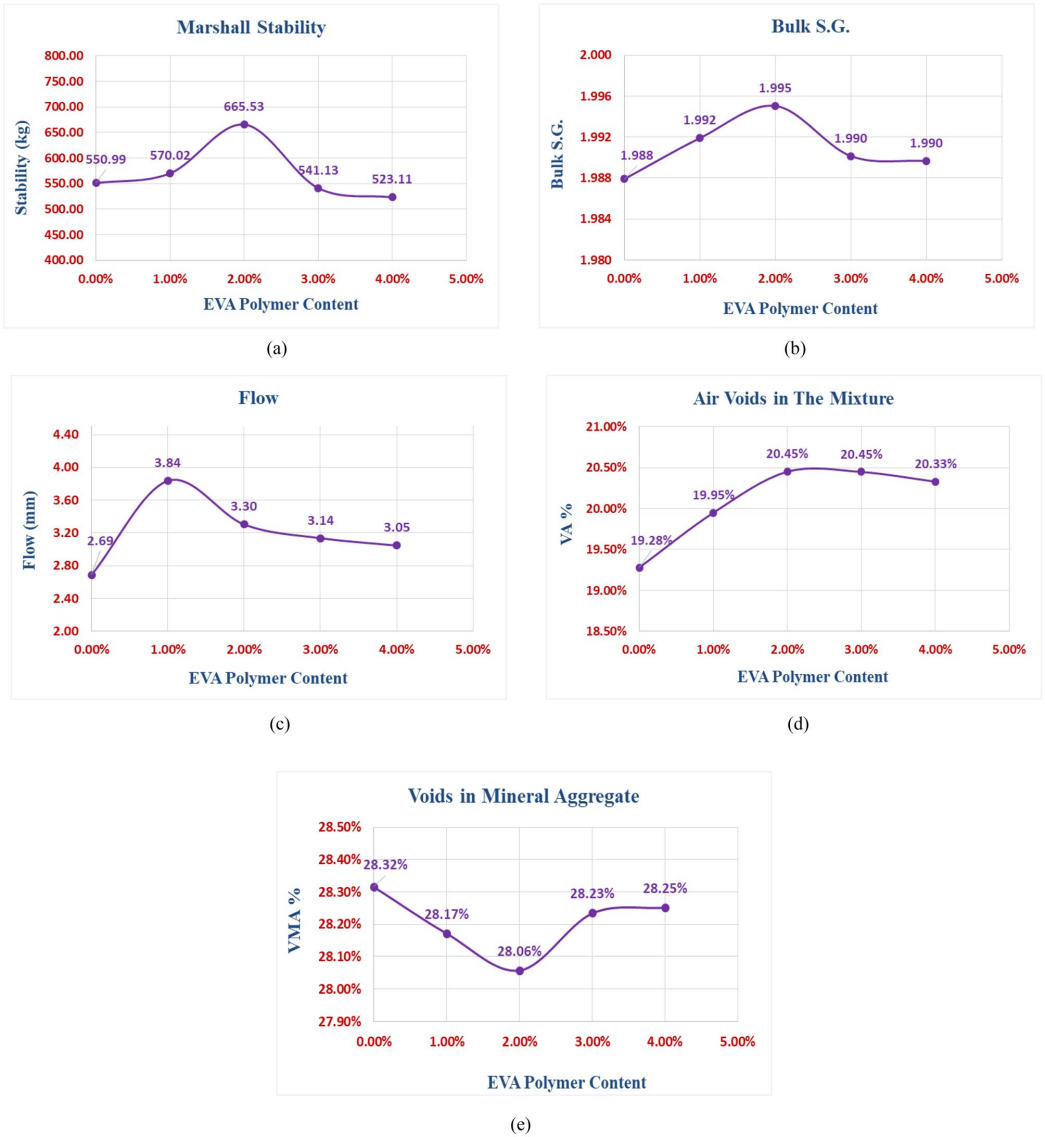
Marshall test results for the EVA modified mixtures. (**a**) Stability results. (**b**) Bulk specific gravity results. (**c**) Flow results. (**d**) Air voids results. (**e**) Voids in mineral aggregate results.

The objective of the third stage was to determine the O.E.C. through Marshall Test results. Figure 9a–e show the relation between the percentages of EVA Polymer content and stability, S.G., flow, VA%, and VMA%, respectively.

The results demonstrated that the Marshall stability increased from 551 kg at 0% EVA polymer to 665.5 kg at 2% EVA polymer, after which the curve went down. The EVA polymer improved the stability by 20.8% compared to the control mixture, which enhancing the asphalt mixture resistance to traffic loads and rutting. The EVA polymer additive contributed to the adhesion ability of the aggregates in the porous asphalt mixture, leading to enhanced mixture performance. It was observed that the bulk specific gravity reached its peak at 2% EVA polymer. From the flow chart, all the modified mixtures with different percentages of EVA polymer met the requirements of NAPA-IS115. Increasing the EVA polymer content led to an increase in the air void percentage, making the asphalt mixture more permeable. Eventually, it was found that the O.E.C. is **2%**, as it gave the highest stability, the highest specific gravity value, and a reasonable flow and air void percentage. Table 7 shows the properties of the OEC porous asphalt mixture.

**Table 7:** The properties of OEC porous asphalt mixture.

Property	Result	(NAPA) requirements for OGFC
Stability (kg)	665.53	500
Flow (mm)	3.3	2–6
Marshall quotient (kg/mm)	201.68	200
Air voids (%)	20.45	> 18
Bulk specific gravity	1.995	–
Voids in mineral aggregate (%)	28.06	–

### Fourth stage

The objective of the fourth stage was to evaluate the OEC porous asphalt mixture and compare between the control mixtures and the modified mixtures through four different performance tests.

#### Binder draindown test

**Figure 10:**
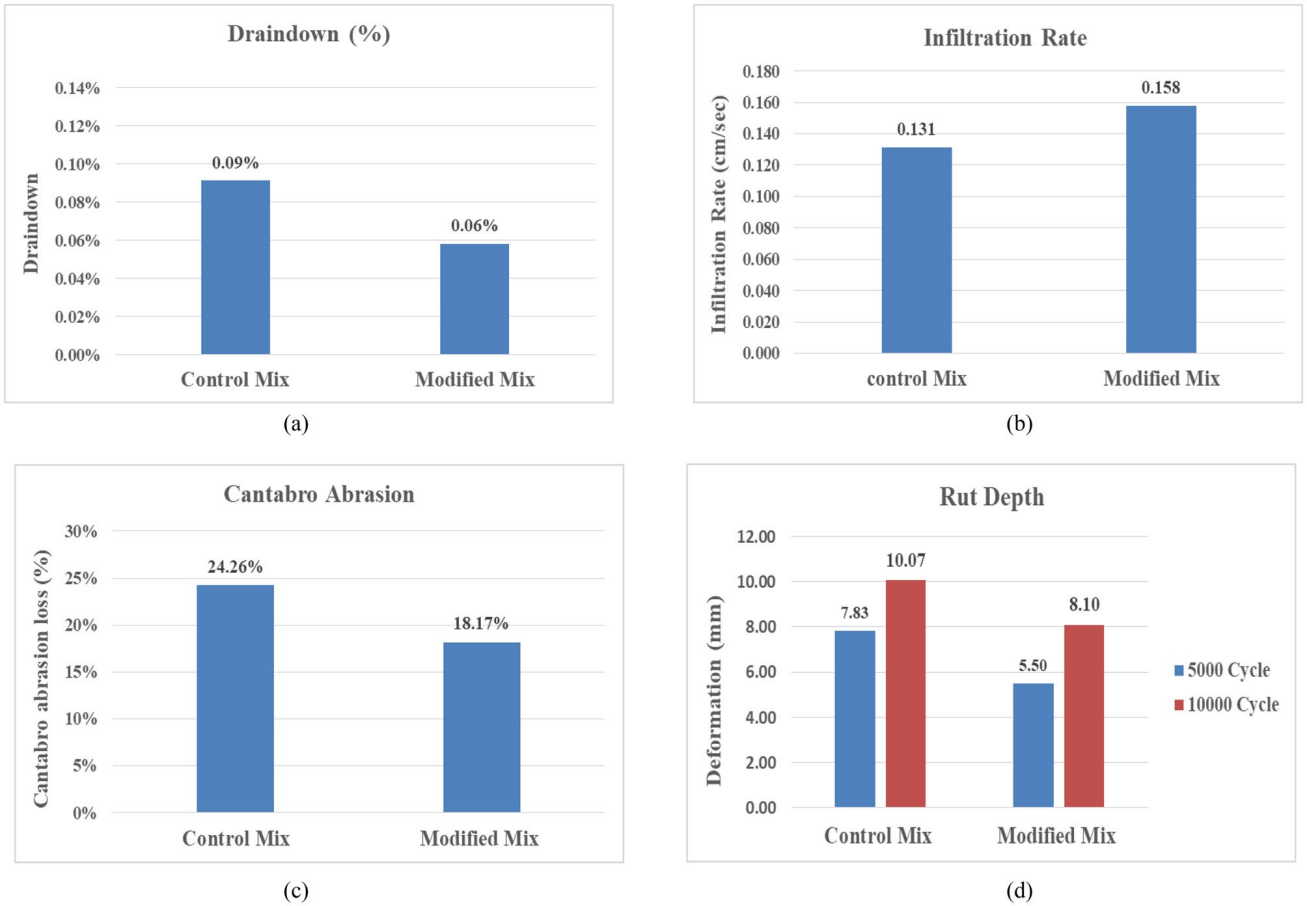
Performance tests results. (**a**) Draindown propotion. (**b**) Infiltration rate. (**c**) Cantabro abrasion loss. (**d**) Hamburg Wheel-Track test (rut depth).

Figure 10a shows the results of the draindown test for both the control mixtures and the EVA-modified mixtures. They both met the NAPA IS115 requirement for draindown ratio, which is 0.3% max^2^. It was found that there was an enhancement by 33.3% for the EVA-modified mixture compared to the control mixture. This occurrence may be attributed to the adhesion ability of EVA-modified bitumen to the aggregates, resulting in a reduction of the draindown ratio, i.e., better draindown resistance.

#### Infiltration rate test

Figure 10b shows the results of the infiltration rate test for both the control mixture and the EVA modified mixture. Both mixtures met the requirement of NAPA IS115 for the permeability of OGFC, which set at a minimum of 0.116 cm/s^2^ and also satisfied the requirements of AAPA, which is 0.058–0.249 cm/s^19^. From the results, the EVA-modified mixtures gave a better infiltration rate, which is favorable for the porous pavement. There was a notable improvement by 20.6% compared to the control mixture. The improvement was a result of the EVA-modified mixture having more air voids than the control mixture.

#### Cantabro abrasion test

**Figure 11:**
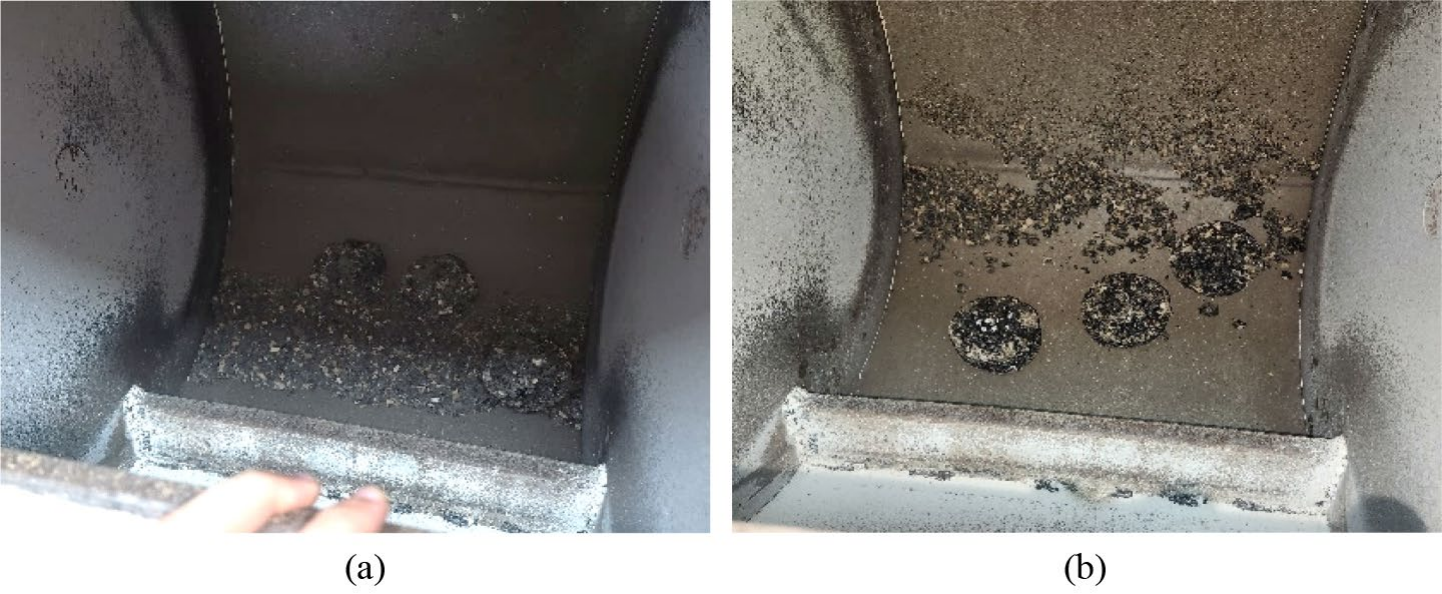
Porous asphalt mixtures after 300 revolutions into Los Angeles machine (**a**) control mixtures, (**b**) modified mixtures.

Figure 10c shows the cantabro abrasion loss percentage for both the control mixtures and EVA-modified mixtures. The EVA-modified mixture met the requirement of NAPA IS115, which is 20% max^2^. However the control mixture failed to meet this requirement. Upon examining the samples subjected to abrasion, it was found that the particles in the control mixtures exhibited greater separation from the mixture compared to the modified mixtures. This observation suggests that the inclusion of EVA-modified bitumen enhanced the adhesion between the aggregate. Consequently, the addition of EVA polymer has the potential to enhance the resistance of porous asphalt mixtures to abrasion. Figure 11 shows the porous asphalt samples at the end of the test.

#### Hamburg wheel-track test

Figure 10d shows the rut depth results (in mm) for both the control mixtures and EVA-modified mixtures after 5000 and 10,000 cycles in the Hamburg Wheel-Tracking Machine. The results indicated that addition of EVA polymer significantly increased the rutting resistance as it decreased the rut depth from 7.83 to 5.50 mm and from 10.07 to 8.10 mm at 5000 cycles and 10,000 cycles, respectively. This occurrence could be attributed to the increased stiffness of the EVA-modified bitumen in comparison to the base bitumen, which made the modified mixture less susceptible to rutting, i.e., more resistant to permanent deformation.

## Conclusion

In this study, different laboratory tests were conducted on the porous asphalt mixtures with different fibers and additives. The main conclusion of this study can be summarized in the following points:

- Cellulose fiber emerges as a more fitting choice for porous asphalt mixtures compared to glass wool fiber, as it gave better performance in the Marshall Test.
- EVA polymer increases the softening point, significantly increases dynamic viscosity, and decreases the penetration values of the bitumen 60/70. It also improves the temperature susceptibility of the bitumen, as the penetration index increased from − 0.358 for the base bitumen to 1.414 for 4% EVA polymer content.
- SEM analysis indicated that EVA polymer well dispersed in the bitumen and made a homogenous structure for all ratios used, 1%, 2%, 3%, and 4%.
- The optimum bitumen content was 4.5% of the total weight for the porous asphalt mixtures with 0.3% cellulose fibers, while the optimum EVA polymer content was 2% of the bitumen weight.
- The addition of EVA polymer increased Marshall stability by 20.8% compared to the control mixtures; however, the Marshall quotient was almost the same.
- The percentage of draindown in the mixtures modified by the EVA polymer decreased by 33.3%, and the infiltration rate increased by 20.6% compared to the control mixtures.
- EVA polymer improves the traffic abrasion resistance of the porous asphalt mixtures, as it decreased the cantabro abrasion loss by 25.1% compared to the control mixtures and enhances the rutting resistance, as it decreased the rut depth at 5000 cycles and 10,000 cycles by 29.8% and 19.7%, respectively.

## Data Availability

The data that support the findings of this study are available from [General Authority for Roads and Bridges and Land Transport (GARBLT)], but restrictions apply to the availability of these data, which were used under license for the current study and so are not publicly available. Data are, however, available from the authors upon reasonable request and with the permission of [GARBLT], https://garb.gov.eg/.

## References

[1] Eisenberg, B., Collins, K., & Smith, D. *Permeable Pavements* (2015).

[2] Prithvi, S., & Kandhal, P. E. Design, Construction, and Maintenance of Open-Graded Asphalt Friction Courses, National Asphalt Pavement Association (NAPA) Information Series 115 (2002).

[3] Gemayel CA, Mamlouk MS (1988). Characterization of hot-mixed open-graded asphalt mixtures. Transp. Res. Rec..

[4] Huber, G. Performance survey on open-graded friction course mixes (2000).

[5] Ruiz A, Alberola R, Pérez F, Sánchez B (1990). Porous asphalt mixtures in Spain. Transp. Res. Rec. J. Transp. Res. Board.

[6] Zhang Z (2020). State-of-the-art of porous asphalt pavement: Experience and considerations of mixture design. Constr. Build. Mater..

[7] Çetin A, Oral G (2022). Performance evaluation of porous asphalt mixtures modified with basalt fiber. Rev. la Constr..

[8] Joohari MI, Aziz NA, Daud NM, Mansor S, Abdul Halim MA (2019). Performance of porous asphalt pavement using clay brick dust as mineral filler. J. Phys. Conf. Ser..

[9] Chaira M (2021). Characteristics of porous asphalt mixture by using a bottom ash boiler as a filler. J. Phys. Conf. Ser..

[10] Basiouny M, Eisa MS, Elsayed E, Elsayed A (2017). Evaluation of porous asphalt mixtures stabilized by cellulose fibers. New York Sci. J..

[11] Zhang W, Li Q, Wang J, Zeng X, Yu B (2024). Evaluation of the effect of different preparation processes on the road performance of thermoplastic resin modified porous asphalt mixtures. Constr. Build. Mater..

[12] Clara E, Barra BS, Teixeira LH, Mikowski A, Hughes GB, Nguyen ML (2022). Influence of polymeric molecular chain structure on the rheological-mechanical behavior of asphalt binders and porous asphalt mixes. Constr. Build. Mater..

[13] Robinson, H. L. Polymers in Asphalt: EBSCOhost. In *Shawbury, Shrewsbury, Shropshire, U.K. Rapra Technol. Ltd.*, vol. 15, no. 11 (2004).

[14] Sengoz B, Isikyakar G (2008). Evaluation of the properties and microstructure of SBS and EVA polymer modified bitumen. Constr. Build. Mater..

[15] Mallick, R. B., Kandhal, P. S., Cooley, L. A., & Watson, D. E. Design, construction, and performance of new-generation open-graded (2000).

[16] M. Application and M. Properties. FL 65 PH Ethylene vinyl acetate copolymer, vol. 178, no. November, pp. 1–2 (2020).

[17] Cooley J, Brown ER, Watson DE (2000). Evaluation of open-graded friction course mixtures containing cellulose fibers. Transp. Res. Rec..

[18] ASTM D1559. Standard test method for resistance to plastic flow of bituminous mixtures using Marshall apparatus. *ASTM International, West Conshohocken, PA*, vol. 5. pp. 1–5 (2018).

[19] A. Australian Asphalt Pavement Association. *National Asphalt Specification*, no. April (2004).

[20] Lu X, Isacsson U (1997). Characterization of styrene-butadiene-styrene polymer modified bitumens—comparison of conventional methods and dynamic mechanical analyses. J. Test. Eval..

[21] Read, D. J., & Whiteoak, M. D. v8_1008 - Shell Bitumen Handbook, no. 7 (2003).

[22] Mazumder M, Ahmed R, Wajahat Ali A, Lee SJ (2018). SEM and ESEM techniques used for analysis of asphalt binder and mixture: A state of the art review. Constr. Build. Mater..

[23] ASTM D 6390. Standard method of test for determination of draindown characteristics in un-compacted asphalt mixtures. *Annu. B. Am. Soc. Test. Mater. ASTM Stand.* 6–8 (1997).

[24] American Society of Testing Material. Standard test for surface infiltration rate of permeable unit pavement systems. *ASTM Int.* pp. 1–5. 10.1520/C1781 (2013).

[25] ASTM-D7064. Standard practice for open-graded friction course (OGFC) mix design. In: Annual book of ASTM standards, *West Conshohocken, PA 19428–2959. United States*, vol. 7, no. Reapproved, pp. 1–7. 10.1520/D7064 (2013).

[26] American Association of State Highway and Transportation Officials. AASHTO T 340 Standard Method of Test for Determining Rutting Susceptibility of Hot Mix Asphalt (HMA) Using the Asphalt Pavement Analyzer (APA), vol. 10, (2023).

[27] AASHTO T 324-11. Standard method of test for Hamburg wheel-track testing of compacted hot mix asphalt (HMA). *Am. Assoc. State Highw. Transp. Off.* (2013).

[28] Ma X, Li Q, Cui YC, Ni AQ (2018). Performance of porous asphalt mixture with various additives. Int. J. Pavement Eng..

